# Molecular docking and in vitro analysis of phytoextracts from B. serrata for antibacterial activities

**DOI:** 10.6026/97320630017667

**Published:** 2021-07-31

**Authors:** Ramsi Vakayil, Murugesan Anbazhagan, Gnanendra Shanmugam, Srinivasan Ramasamy, Maghimaa Mathanmohun

**Affiliations:** 1 Department of Microbiology, Muthayammal College of Arts and Science, Rasipuram, Namakkal, Tamilnadu, India; 2 Department of Botany, Government Arts and Science College, Thiruvannamalai - 606603. Tamilnadu, India; 3 Bioinformatics Division, Origene Biosolutions, Salem 16. Tamilnadu, India; 4 Member secretary, Tamil Nadu State Council for Science & Technology, Chennai,Tamilnadu, India

**Keywords:** Plant extract, GCMS, bioactive compounds, Molecular docking

## Abstract

The bioactives of Boswellia serrata have a role in ulcer healing therapies. Eleven bioactive compounds were obtained by GC-MS among which Cholan-24-oic acid, 3,12-bis (acetyl oxy) has a high molecular weight of 490.6719 with a retention time of 26.729. Twenty wound samples were collected aseptically from the labs and hospitals in and around the Namakkal districts of Tamilnadu, India. The antibacterial potential of E.coli showed a maximum inhibition of 27 mm against Tetracycline at 30µg. The ethanolic extract of the B. serrata shows a susceptibility of 19mm towards E. coli at 60µg concentration in MIC. Molecular docking results show the binding energy of Cholan-24-oic acid, 3,12-bis(acetyloxy) -8.6 (kcal/mol) followed by Pyrene, hexadecahydro- -6.7 (kcal/mol), and 5(1H)- Azulenone, 2,4,6,7,8,8a-hexahydro-3,8-dimethyl-4-(1-methylethylidene)-, (8S-cis)- 6.4 (kcal/mol) for further consideration.

## Background:

The diseased field is replaced by rejuvenating content due to the result of the immune system and for attaining new epithelialization [1]. Infection ranges from a simple wound to septicemia and the pathogenicity is treated with novel drugs from plants that fulfill the targets [2]. In spiritual formalities, the Boswellia groves and their lubricant are used as bouquets [3]. The resin of the Boswellia serrata acts like a lytic of tumors (ganglions healer) [4]. Leukopenic power helps in treating autoimmune and genetic disorders it is also known as olibanum. The ingredients in the Boswellia serrata supports and quickens not only the repairing process but also urge the formation of the strengthened scars [5]. MAE usage of solvents is very minimal, in-expensive, low consumption of time, high yield, best for extracting lipids and glycans from the various origin so it is entitled as 'green technique [6]. Therefore, it is of interest to document the molecular docking and in vitro analysis of phytoextracts with B.serrata for antibacterial potentials.

## Materials & Methods:

### Collection of resins:

Fresh bronzed or bottle green resins brought from the local market of Rasipuram are then washed in Milli-Q water air-dried and grounded into powder using an electric mixer.

### Extraction:

For extraction the weighed powdered resin was mixed with the solvents such as ethanol and aqueous at a different ratio in a 250 ml Erlenmeyer flask and placed over the circulating disk in the oven [7]. Parameters like temperature, time are maintained as per the protocol. Filtered aqueous phase air-dried as per the formula the dry weight of each crude is examined and maintained the crude at -5 degree C for future process.

### Gas Chromatography-Mass Spectrometry (GC–MS):

The system used is Agilent GC 7890A/ gas chromatograph MS detector MS5975C, US and samples dissolved in dichloromethane Gas chromatography linked to a mass spectrometer (GC0-MS) equipped with fused silica capillary column and an Agilent DB5MS, (Column Length: 30m/0.25mm internal dia/0.25micron film thickness.

### Wound and skin samples:

Encircling the Namakkal district approximately twenty samples were collected from the different ulcer, diabetic and sore patients [8,9]. Using sterile swabs swabbed the pus, wound, and other exudates then which are packed aseptically in a transport media containing polypropylene container, sealed and marked the history of the specimen and stored at -4 degree C brought to our laboratory for research work, received from various sophisticated labs and hospitals.

### Isolation and identification:

In various selective media, the collected commensals are inoculated and incubated at room temperature. After 24 hrs, the natural edges, texture color, and odor of the colonies are visualized, for further phenotypic identification a few drops of primary stain sprinkled on the smear in the slide then washed using H2O after few seconds the slide was flooded with mordant (iodine) mean-while a quick water wash was done. A few minutes later, the slide is rinsed with the decolorizer (alcohol) and then the slide is shown under the tap water. At last gram +ve and gram-ve are identified by the counterstain safranin which is spread on the slide and excess stains are removed by showering the smear in water and droplets are isolated by wrapping in soft tissue paper [10]. Gaseous bubble formation, pink, violet, purple cherry red color appearance, and production of nonorganic acids are the positive signs in biochemical tests of some pathogens to identify their metabolic and enzymatic characteristics [11].

### Antibiotic sensitivity:

Isolates of gram-positive and gram-negative spread uniformly all over the MHA plate, along with a circular disk loaded with antibiotics are kept aseptically in the center of the plate and then incubated. Simultaneously, [10] another plate loaded with antibiotic disks without the inoculation of the pathogen was maintained as control kept for incubation and observed. After 24 hrs, MDR, PR, and sensitivity against a broad spectrum of antibiotics are measured.

### Antibacterial activity:

A sterile cork Borell of 6 mm is used to make a well on the MHA plate for diffusion [12], [13 - check with author]. Then crude of aqueous and ethanol at different concentrations (20µg, 40µg, and 60µg) is loaded on the well to examine the antagonistic activity of the crude against the inoculated wound gram+ve and gram-ve isolates and kept for incubation. After 24 hrs, the zone of inhibition or minimal inhibitory concentration is noted by observing the halo around the well.

### Molecular docking:

Small preliminary work is done for selecting the protein molecule by downloading (www.rcsb.org) or PDB format. Editing is done in the format via pymol or word pad tool [14]. The protein chain in the document begins with the letter ''TER''and this shows the chain is terminated and the file is saved, ready for docking [15]. For the execution of docking install "autodock suite-4.2.5.1- i86Windows.exe downloaded from the website (http://autodock.scripps.edu/) Mol soft and chimera is used to draw the ligand structures. The molecules, ligands, and amino acid interaction and their energies are predicted by the software tools [16] until they are present in the grid box. The active site, binding site, and other essential regions of the molecules are predicted after setting the grid box. All 'PDP' files of protein and ligands are moved into the 'work folder' for further execution of docking.

### Ligand preparation:

The GC-MS identified bioactive compounds of the plant extract B. Serrata were chosen for the current study using i) Ethyl 2-chloro propionate ii) alpha-Asarone iii) 5-Dodecyne iv) 5-Isopropenyl-2-methyl-7-oxabicyclo v) o-Mentha-1(7),8-dien-3-ol vi) Carbonic acid, 2-chloroethyl 2,2,2-trichloroethyl ester vi)Benzene, 1-[(2-chloroethyl)sulfonyl 2-nitro vii) 3-chloro-4-nitrophenol viii) Cholan-24-oic acid, 3,12-bis(acetyloxy) ix) Pyrene, hexadecahydro- viii) 5(1H)-Azulenone, 2,4,6,7,8,8a-hexahydro-3,8-dimethyl-4-(1-methylethylidene)-, (8S-cis)- and the antibiotic reference drugs used for molecular docking were Gentamycin, Meropenem, Tetracyclin, and Vancomycin. The threedimensional (3D) structures of all the selected cyano compounds were retrieved from the pub chem compound database https://pubch em.ncbi.nlm.nih.gov/ in the SDF file which was then converted into PDB format for docking study [17 - check with author,^18^].

## Results and Discussion:

**Figure 1:**
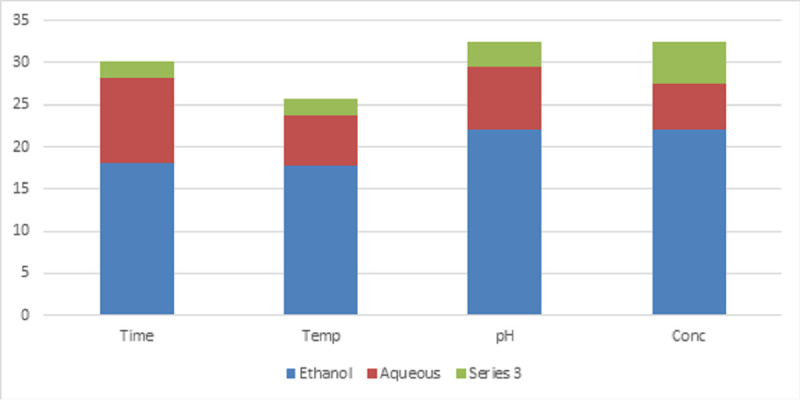
Yield of the crude ethanol and the aqueous

**Figure 2:**
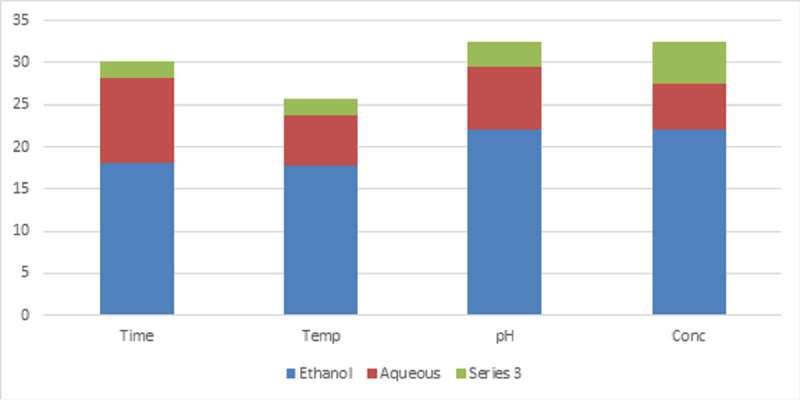
AmPC E. coli - CID 21140628 Docking Pose & Interaction Plot (-8.5 Kcal/mol)

**Figure 5:**
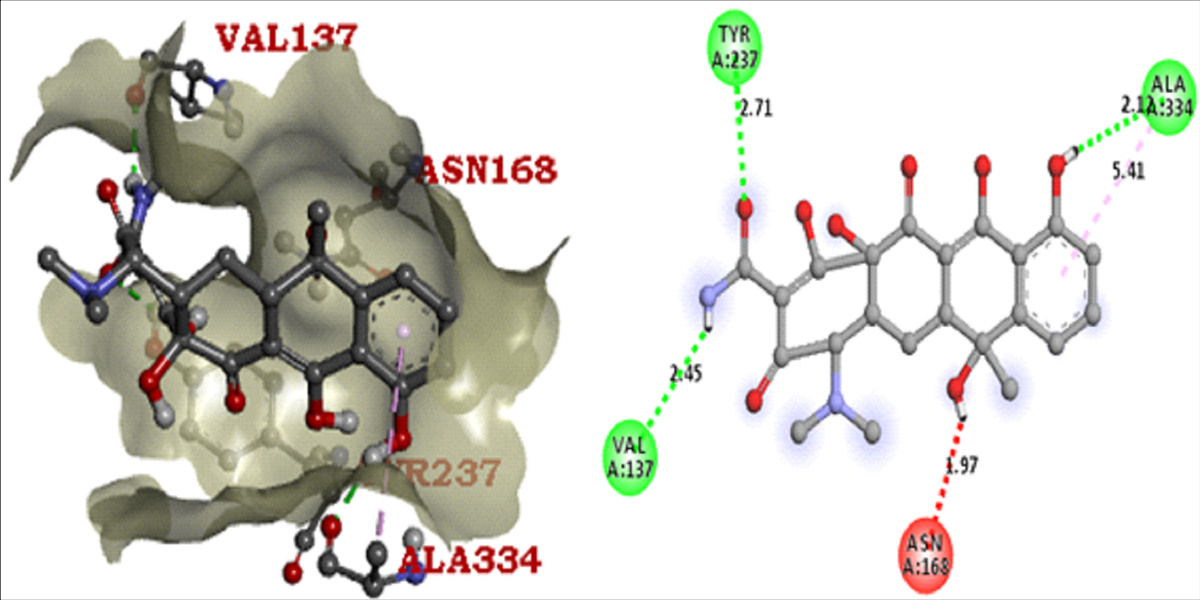
AmPC E. coli- CID 54675776 Docking Pose & Interaction Plot (-8.0 Kcal/mol)

The colony morphology of the pathogens wound isolates is cohesive, raised off–white, mucus and shiny texture like colonies are observed in the MSA, nutrient agar, blood agar, EMB agar and Mcconkey agar this indicates the isolates are E.coli, and S.aureus, whether they are gram +ve or gram-ve is identified along with the biochemical study they are briefly described in Table 1(see PDF). Among the broad spectrum of antibiotics such as Tetracycline, Meropenem, Vancomycin, and Gentamycin, the gram -ve bacteria E.coli show resistance to tetracycline and vancomycin because the zone of inhibition is in the range of 6-14mm. But in the case of S.aureus, it also shows resistance to vancomycin, because the ZOI is 16 mm. Then the antibiogram profile with other antibiotics is listed in Table 2(see PDF). In MIC the ethanolic and aqueous extract of B. serrata resins show susceptibility towards gram-negative bacteria of E.coli with a zone of inhibition of 19mm at 60µg concentration but S. aureus showed a zone of inhibition in the range of 15 and 17mm. The other concentrations and their minimal inhibitory concentration level are shown in Table 3(see PDF). The crude of the resin obtained by ethanolic extraction shows high yield than compared with the aqueous extraction by maintaining different parameters like time (5, 10, 15, 20),temperature (200W, 300W, 500W, and 700W), pH (6, 7, 8, 9)concentration (100, 100, 100, 100). Therefore, the crude ethanolic resin obtained at 15minutes at the temperature of 700W provides a good yield and is shown in Figure 1. The yield is determined by implementing the dry weight formula shown below: Dry wt% =Wt. of the dry extract x 100/ Wt of the resin PWD The GC-MS analysis explored eleven bioactive compounds in the ethanolic extract. The molecular formula, molecular weight, retention time and area % of the compounds are presented in Table 4(see PDF). Among the observed bioactive compounds the Cholan-24-oic acid, 3,12 bis(acetyloxy) show a binding affinity of -8 (kcal/mol) and the reference antibiotic tetracycline also has the same binding affinity (-8 (kcal/mol) with ligand Ampc E. coli. Arg220, Thr332, Asn359, Asn362, Leu135, Tyr237, Ala334 are the active site residues in the beta-lactamase protein molecule. The binding score and 3D graphical structure are all shown below with their CID 21140628, CID75524, CID 91735354, and CID 54675776 (Tables 5 to 6 - see PDF and Figure 2-Figure 5). The ingredients which are having most effective tumor lysing ache solving WBC production minimizing, fungal resisting inflammation controlling bursal complication resolving types available in saturated forms these are all obtained as per the international protocol experimentally and inhibitory effect explored after treatment with GCMS and docking almost all systems of the physiology CVS, rheumatic, RS,COPD, GI, IBS, CNS, PN along with these especially in the RS very many ailments like genetical, congenital, geriatrics pediatrics, youths (infertility) are all under its control. Genetical hypogonadism, geriatrics, sexual disorders, pediatrics turner's syndrome [19]. In cosmetology, the bioactive compounds of B. serrata are helpful in the management of hair loss and diseases of the nails [20,21].

## Conclusion:

We show the good binding features of the bioactive compound as Cholan-24-oic acid, 3,12 bis(acetyloxy) from B. serrata with AmpC for further consideration in the context of antibacterial potential and wound healing.

## Figures and Tables

**Figure 3:**
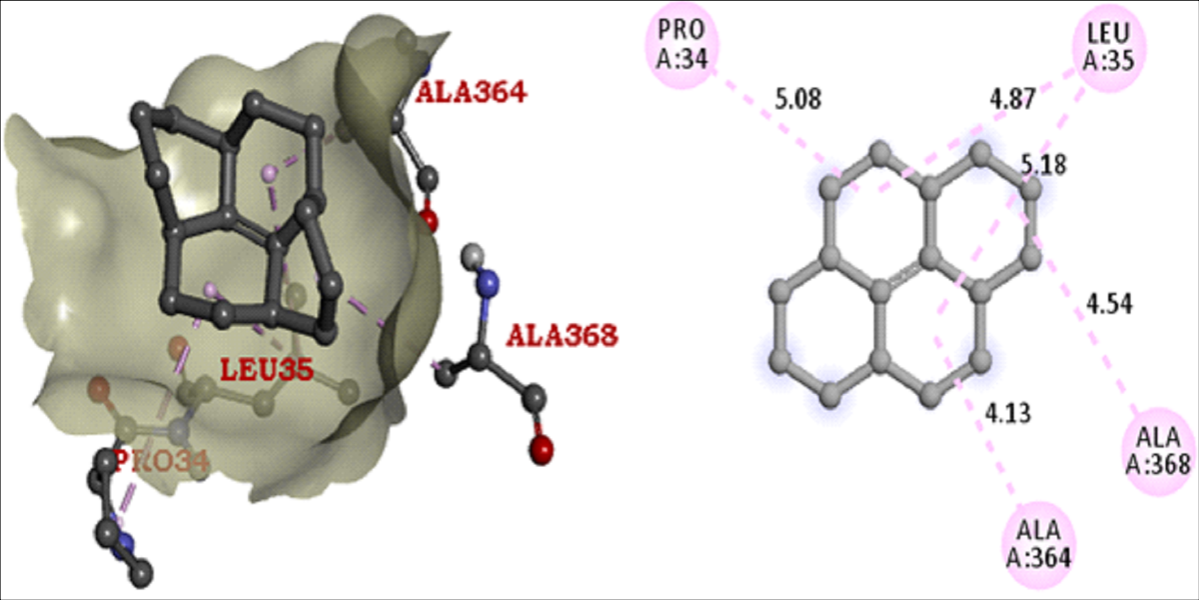
AmPC E. coli - CID 75524 Docking Pose & Interaction Plot (-6.7 Kcal/mol)

**Figure 4:**
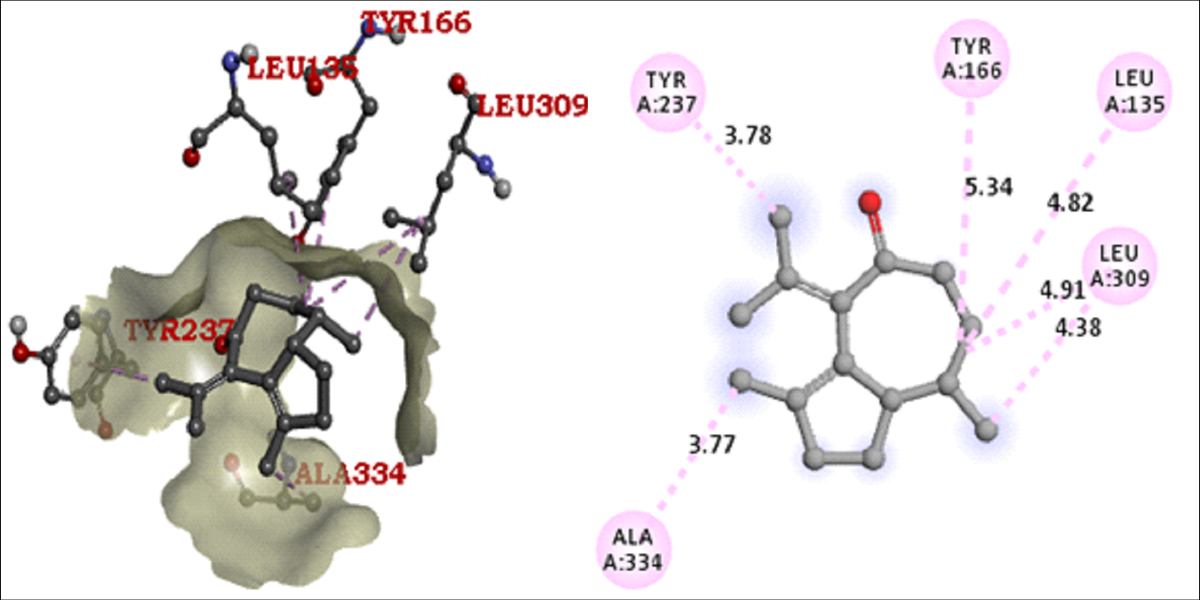
AmPC E. coli - CID 91735354 Docking Pose & Interaction Plot (-6.4 Kcal/mol)

## References

[3] Haroyan A (2018). BMC Complementary and Alternative Medicine.

[4] Notarnicola A (2016). International Journal of Immunopathology and Pharmacology.

[5] Liang M (2019). Journal of Biomedical Materials Research Part B: Applied Biomaterials.

[6] Song R (2019). International journal of biological macromolecules.

[7] Ragab TIM (2019). International journal of biological macromolecules.

[10] Haque S (2019). Mymensingh Med J.

[11] http://mbimph.com/index.php/UPJOZ/article/view/1421.

[12] Maghimaa M, Alharbi SA (2020). Journal of Photochemistry and Photobiology B: Biology.

[14] Burley S (2019). Nucleic Acids Res.

[15] Grither WR, Longmore GD (2018). Proceedings of the National Academy of Sciences.

[20] Beringer A, Miossec P (2019). Nature Reviews Rheumatology.

[21] Karimifar M (2017). Clinical rheumatology.

